# A Case of Bilateral Inflammatory Breast Cancer

**DOI:** 10.7759/cureus.40101

**Published:** 2023-06-07

**Authors:** Sydney Levy, Mariam Hanna

**Affiliations:** 1 Department of Radiology, University of Florida College of Medicine, Gainesville, USA

**Keywords:** diagnostic imaging, metastatic breast cancer, mammography, magnetic resonance imaging, inflammatory breast cancer, breast imaging

## Abstract

Inflammatory breast cancer (IBC) is an aggressive form of breast carcinoma. Bilateral occurrences of IBC within a short time frame are rare, particularly without significant surgical intervention. This case presents a patient with contralateral recurrence of IBC less than a year after the initial diagnosis.

A 39-year-old female was diagnosed with stage IV inflammatory breast cancer in her left breast. Less than a year later, extensive disease was found in her right breast. The patient had received incomplete treatment for the left IBC due to barriers to accessing care. Imaging confirmed the presence of inflammatory breast cancer in the contralateral breast, along with regional adenopathy and metastases. The patient began a chemotherapy regimen similar to her previous treatment.

This case highlights the uncommon occurrence of contralateral recurrence of IBC and the hypothesized mechanism of lymphatic spread, suggesting local metastasis rather than a new primary tumor. The patient’s incomplete treatment and lack of surgical intervention likely contributed to the development of contralateral IBC. The case underscores the importance of magnetic resonance imaging (MRI) in evaluating soft tissue and lymphatic changes in IBC. Barriers to care negatively impact prognosis, emphasizing the need for timely follow-up, diagnostic imaging, and oncologic therapy for successful treatment.

## Introduction

Inflammatory breast cancer (IBC) is a rare, yet highly aggressive subtype of locally advanced breast carcinoma, with a median overall survival rate of less than four years, even with new, multimodality treatment options [1]. The typical presentation of IBC involves the rapid onset of unilateral breast erythema, edema, and peau d’orange [1,2]. Early diagnosis of IBC has proven to be a challenge, due to initial clinical symptoms being similar to those of acute mastitis or abscess. Clinicopathological diagnosis requires an interprofessional approach, beginning with radiographic localization of the suspicious mass through a diagnostic mammogram. Subsequent ultrasound is performed to further characterize the suspicious mass and regional lymph nodes and serve as a guide for core needle biopsy [2]. The hallmark of IBC histopathology is the invasion of the dermal lymphatic system, and this is required to confirm the diagnosis [1].

The development of contralateral IBC at or soon after primary diagnosis is relatively rare, and there is a paucity of definitive cases of bilateral inflammatory breast cancer that have been described in the literature. One recent case report describes a postmenopausal patient who presented for hip pain and was incidentally found to have advanced metastatic bilateral inflammatory breast cancer on computed tomography (CT) and destructive osseous lesions, which had caused a pathological acetabular fracture [3]. Given that distant metastases were present at diagnosis in this patient, the pattern and chronicity of metastatic spread is unknown, unlike the case we describe in this report where the contralateral breast and axilla were the primary sites of reoccurrence. Notably, the patient in the aforementioned case report was found to be estrogen receptor (ER)+, progesterone receptor (PR)+, and human epidermal growth factor receptor 2 (HER2)+, unlike our patient who was only HER2+. ER positivity is associated with a better prognosis due to a more favorable response to therapy [4].

Diffuse lymphatic involvement and subsequent obstruction are implicated in the high degree of distant metastasis in IBC [5], as in the aforementioned case report. This can be extrapolated to the mechanism by which inflammatory breast cancer can spread to the contralateral breast. A single-center retrospective review published in Breast Oncology in 2021 analyzed the frequency of contralateral axillary metastasis (CAM) in patients with unilateral IBC and found that CAM was present in 8.3% of patients at presentation and was best assessed with ultrasound and positron emission tomography (PET) imaging [6]. This is compounded by the fact that IBC in particular is usually advanced (stage IV) at initial diagnosis due to delay in identification that occurs due to both medical and socioeconomic challenges, and this increases the likelihood of more extensive lymphatic invasion and spread at first presentation.

Due to the difficulty of tissue compression, in combination with diffuse tissue edema and trabecular thickening in IBC, mammography is limited in its ability to detect primary breast lesions [2]. For this reason, magnetic resonance imaging (MRI) is the preferred imaging modality to delineate disease extent and has the highest accuracy for detecting primary parenchymal breast lesions in IBC patients [2]. Multiple small, confluent, heterogeneously enhancing masses and global skin thickening are key MRI features of IBC [2], and in addition to its diagnostic value, MRI is often used to monitor response to chemotherapy.

## Case presentation

A 39-year-old female presented with stage IV (T4N1M1) inflammatory breast cancer of the left breast, initially diagnosed in June 2021. She underwent a punch biopsy of the skin, which demonstrated adenocarcinoma with dermal lymphatic invasion characteristic of IBC. Initial staging studies revealed bilateral pathologic adenopathy along with multiple hepatic lesions consistent with metastasis, along with left pleural effusion on CT in July 2021. Immunohistochemical analysis demonstrated that the sample was ER-, PR-, and HER2 Neu 3+, with Ki67 of 76%. Genetic testing demonstrated the PALB2 variant of uncertain significance (VUS), which is a gene that encodes a BRCA2-interacting protein and has recently been shown to increase the risk of developing breast cancer [7].

She was initiated on THP (docetaxel/trastuzumab/pertuzumab) on 7/22/21, 8/12/21, 9/13/21, and 12/3/21, completing a total of four cycles of treatment before being lost to follow-up due to transportation and communication difficulties. Of note, CT obtained in January 2022 showed the resolution of all visible diseases. She presented to the emergency department (ED) in August 2022 for worsening breast pain, axillary adenopathy, and new skin changes in her right breast. A skin biopsy was performed, and surgical pathology showed poorly differentiated carcinoma present within dermal lymphatic spaces. Immunohistochemistry demonstrated ER-, PR-, and HER2+ cells. Recommendations were made for PET/CT and restaging of her cancer, but she was unable to complete imaging studies due to similar transportation issues for several months until December when MRI was obtained.

**Figure 1:**
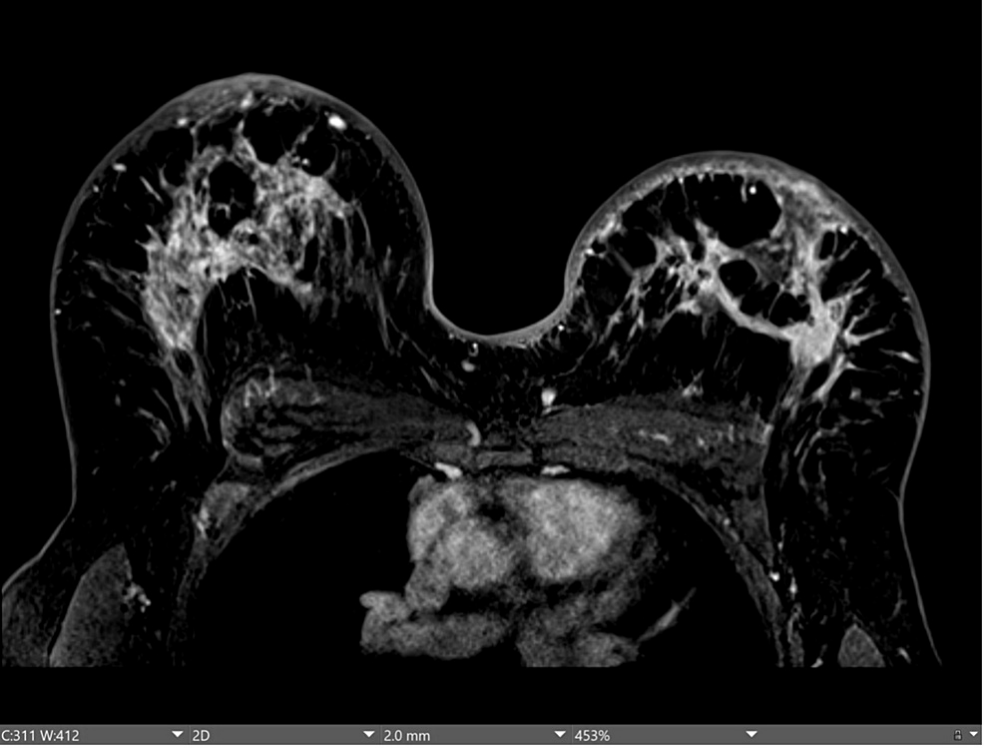
T1-weighted axial contrast-enhanced MRI demonstrating skin thickening of the right and left breasts demonstrating bilateral breast masses and abnormal enhancement. MRI: magnetic resonance imaging

**Figure 2:**
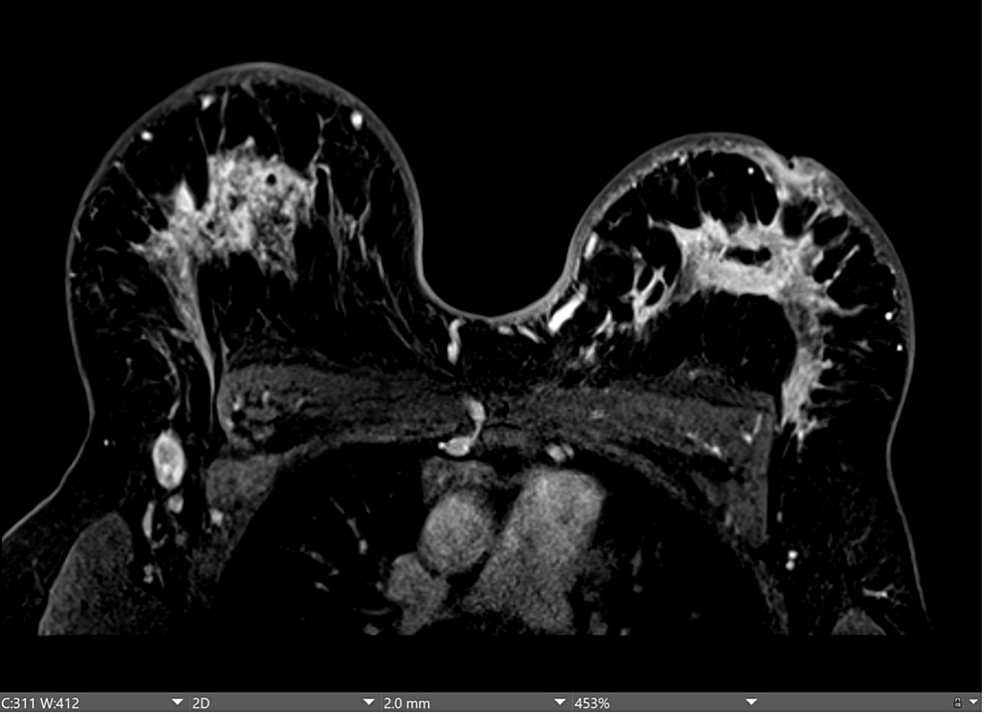
T1-weighted axial contrast-enhanced MRI demonstrating abnormal right axillary lymph node demonstrating rounded morphology. Skin thickening of the right and left breasts demonstrating bilateral breast masses and abnormal enhancement. MRI: magnetic resonance imaging

MRI of the bilateral breasts with and without contrast was notable for moderate and symmetric enhancement with diffuse skin thickening and edema involving both the right and left breasts, with bilateral nipple-areolar complex retraction. Patchy areas of non-mass enhancement were noted in the upper central aspect of the right breast, with nodularity noted in the retroareolar region. Enhancement extended to the pectoralis muscles bilaterally. There were multiple enlarged right axillary lymph nodes, the largest measuring 2.8 × 1.5 cm in size with a cortical thickness of 7 mm. An additional enlarged node was present slightly more superiorly measuring 1.8 × 1.1 cm in size with abnormal morphology and effacement of the normal fatty hilum. No left axillary or internal mammary lymphadenopathy was noted. Right breast findings were highly suspicious for inflammatory breast cancer (Figures 1, 2).

**Figure 3:**
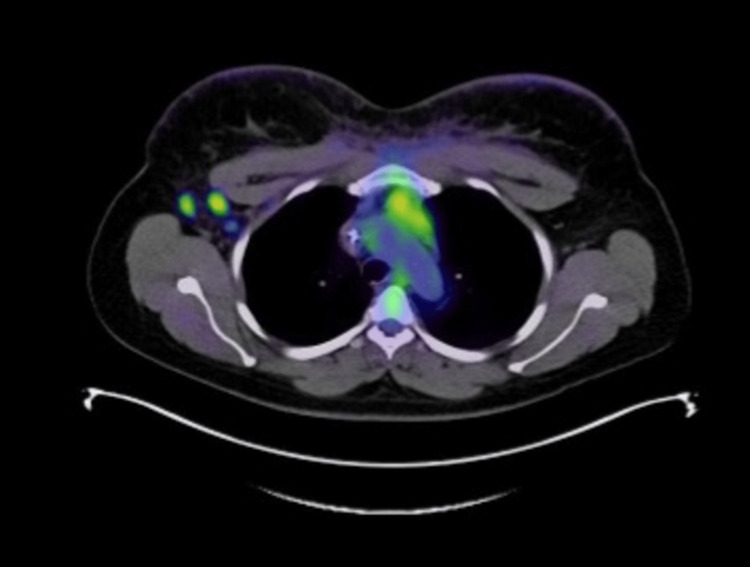
Axial PET/CT image demonstrating hypermetabolic uptake in the right axilla. PET: positron emission tomography, CT: computed tomography

**Figure 4:**
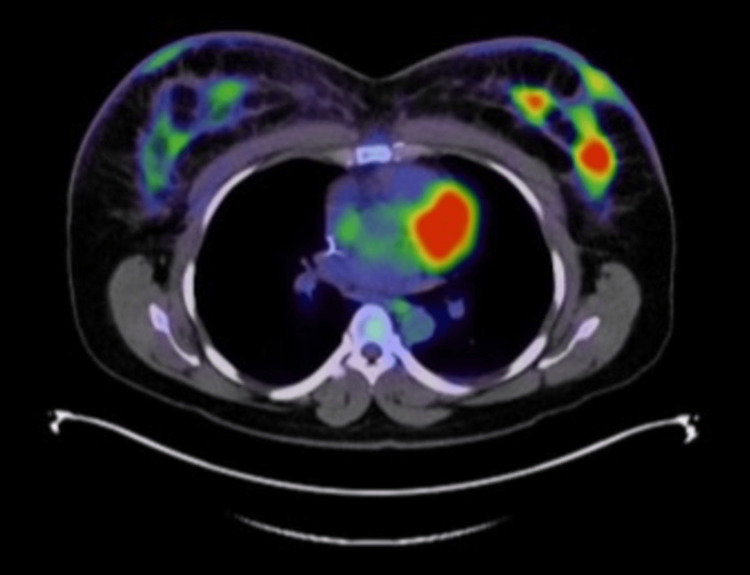
Axial PET/CT image demonstrating hypermetabolic uptake in the right and left breasts consistent with known bilateral breast cancer/malignancy. PET: positron emission tomography, CT: computed tomography

**Figure 5:**
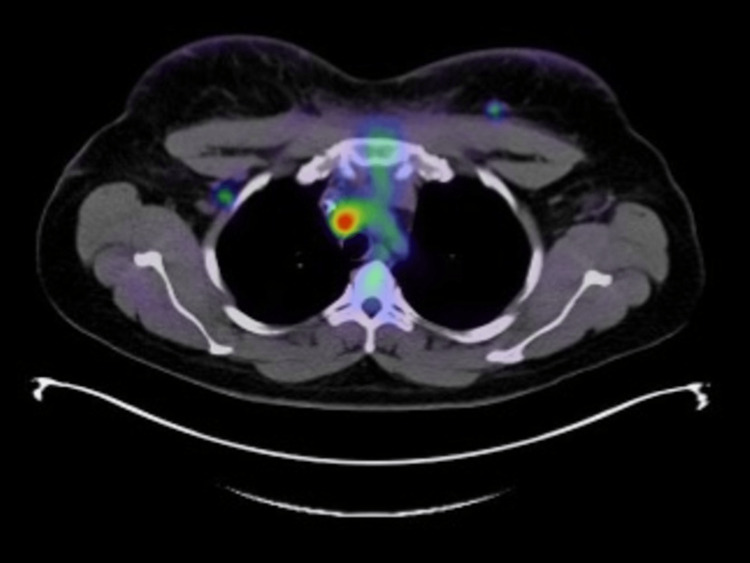
Axial PET/CT image demonstrating hypermetabolic uptake in the superior mediastinum. PET: positron emission tomography, CT: computed tomography

PET/CT demonstrated uptake in both breasts and skin, consistent with inflammatory breast cancer with regional adenopathy, particularly in the right axilla and subpectoral muscle zones (Figures 3, 4). Mediastinal adenopathy was present (Figure 5), consistent with metastasis, in addition to increased uptake in the right ovary and several vertebral bodies.

At a follow-up visit with medical oncology on 12/23/2022, the patient’s visual analog pain score was noted to be 8/10, present in bilateral breasts, only alleviated by opiate pain medications. Given the incurable (stage IV) nature of her disease, sites of metastasis previously noted on initial PET/CT, and her prior success with THP therapy, to which she had a clinical complete response despite not receiving therapy in optimal frequency or duration, the decision was made to reinitiate a similar regimen after obtaining baseline testing including an echocardiogram. Pain management strategies were discussed and initiated prior to starting chemotherapy.

## Discussion

Very few cases of bilateral IBC have been described in the literature. One case report describes a recurrent ipsilateral as well as metastatic contralateral IBC 19 months after the treatment of the primary cancer [8]. A hypothesized mechanism of contralateral spread of IBC is the formation of new lymphatic connections that cross the median plane, secondary to obstruction of lymphatic flow to the axilla in the original side where the primary cancer developed, either due to disruption from modified radical mastectomy or destruction by the cancer itself. This supports the likelihood of local metastasis rather than a new primary tumor. Using data from a population-based registry in the National Cancer Institute’s Surveillance, Epidemiology, and End Results (SEER) program, Schairer et al. [9] found that contralateral breast cancers occurring within the first 2-23 months of first IBC diagnosis may more likely be recurrent/metastatic disease, whereas those occurring two or more years after diagnosis were more frequently independent primary cancers, regardless of age and hormone receptor status.

The diagnosis of IBC using conventional imaging with mammography and ultrasound has proven to be a challenge due to the nonspecific nature of the characteristic findings. The emergence of breast MRI with IV contrast has greatly enhanced the detection and detailed evaluation of IBC lesions and local invasion of lymphatics or chest wall structures [2]. Although the patient in this case presented with extensive disease at the time of imaging, MRI at the time of both primary diagnosis and contralateral disease recurrence was utilized in order to direct further workup with biopsy and guide treatment decisions based on the extent of disease.

## Conclusions

This case describes a recurrence of IBC in the contralateral breast, less than one year following the initial diagnosis. The patient in this case study underwent incomplete chemotherapy and did not undergo any surgical intervention for the primary cancer. Therefore, the contralateral IBC that developed in the following months may have been a result of the destruction and disruption of lymphatics caused by the primary tumor. This case report emphasizes the diagnostic utility of MRI in evaluating the distinct soft tissue and lymphatic changes in IBC. Barriers to care are a negative prognostic indicator, especially for aggressive malignancies, and these factors greatly hinder the prompt clinical follow-up, completion of diagnostic imaging, and timely initiation of oncologic therapy, all of which are necessary for successful treatment.
